# Exploring E-health-literacy-related behaviours and information-seeking practices among university students in Saudi Arabia: a 2024 cross-sectional survey

**DOI:** 10.3389/fpubh.2026.1893795

**Published:** 2026-10-28

**Authors:** Haitham Alzghaibi, Manahil Alfuraydan, Ola Mousa, Munerah Almulhem

**Affiliations:** 1 Department of Health Informatics, College of Applied Medical Sciences, Qassim University, Buraydah, Saudi Arabia; 2 Department of Health Information Management and Technology, College of Applied Medical Sciences, King Fisal University, Al-Ahsa, Saudi Arabia; 3 Department of Women Health, Faculty of Nursing, Minia University, Minya, Egypt; 4 Department of Public Health, College of Applied Medical Sciences, King Fisal University, Al-Ahasa, Saudi Arabia

**Keywords:** AI health tools, digital health literacy-related behaviours, digital health trust, health information seeking behaviour, infodemic, MOH digital services, Saudi Arabia, self-diagnosis

## Abstract

**Background:**

The COVID-19 pandemic accelerated reliance on digital health information, while simultaneously intensifying exposure to misinformation. In this context, digital health literacy and information-seeking behaviour have become critical for informed decision-making, particularly among university students who are highly engaged in digital environments.

**Aim:**

This study aimed to investigate digital-health-literacy-related behaviours, health information-seeking behaviour, and the use of official and AI-supported digital health tools among university students in Saudi Arabia in 2024.

**Methods:**

A cross-sectional survey was conducted between April and May 2024 among university students across Saudi Arabia using a convenience sampling approach. Data were collected via an online questionnaire assessing demographic characteristics, digital health engagement, information-seeking behaviour, verification practices, and perceptions of Ministry of Health (MOH) digital services and AI-based tools. Descriptive statistics and non-parametric analyses were performed using SPSS and R.

**Results:**

A total of 506 students participated. However, self-diagnosis behaviours were less frequent. Participants reported using multiple digital platforms simultaneously, with search engines and video-based platforms being the most dominant. Verification behaviour was common, involving both formal sources (e.g., government platforms and scientific literature) and informal sources (e.g., family and peers). Familiarity with established MOH services was relatively high, Trust in AI-supported tools was moderate, with most participants preferring to confirm digital outputs with healthcare professionals.

**Conclusion:**

University students demonstrate active but cautious engagement with digital health information. Their behaviour is characterised by multi-platform use, layered verification, and continued reliance on professional healthcare. These findings may inform efforts to strengthen digital-health-literacy-related behaviours and improve trust in official digital health services to support safer health decision-making in current digital health environments.

## Introduction

A novel coronavirus (COVID-19) has emerged as a major global health challenge due to its rapid transmission and uncertainty regarding the disease (36; 37). Additionally, COVID-19 has a significant impact on physical health, as well as an intensification of both accurate and inaccurate information on digital platforms (1). Due to the wide reach of social media and the Internet, rumors and unreliable information are spread easily (2). People face a threat of infodemic (short for ‘information epidemic’) due to an overabundance of information on COVID-19. According to the WHO, fighting infodemics requires reporting misinformation to authorities, disseminating accurate information, and developing action plans (3). Health literacy is a crucial concept in this context (4).

Developing health literacy requires the ability to find, understand, appraise, and apply health information across health care, disease prevention, and health promotion domains (5). Digital health literacy (DHL) or eHealth literacy refers to the ability for people to search electronic sources for information and critically review it to make health-related decisions. In the time of COVID-19 pandemic, when health information is mainly available by digital means, health literacy is a crucial concept (6). People must learn how to navigate these information environments and process and filter information in order to make health decisions. People experience DHL differently depending on their literacy level, socioeconomic status, health issues, motivation for seeking online information, and familiarity with technology (7). Health literacy support is essential for patients with limited skills in understanding and assessing information in order to improve their health (8).

The process of seeking and obtaining health or healthcare information is known as health information seeking behaviour (HISB) (9, 10). Searching for health and wellness related information can include finding information about specific illnesses, treatments, or medications (11). A wide range of online and offline sources can be used to gather health information, including social media, television and radio programs, scientific papers, books, online health information websites, healthcare professionals, and family members (12).

Due to the increasing availability of digital devices and the internet, individuals usually seek health information online. Study results from a national representative sample of 1,277 adults in Greece showed that 90% of university students used the internet for health information search and 85.4% found the information provided by websites to be adequate (13). According to a study in Turkey, adolescent health literacy correlated positively with health-promoting behaviours and health responsibility (14). Health literacy was also positively related to health awareness and satisfaction among 1,055 Turkish adult patients in primary healthcare (15). E-Health’s quality of information is also crucial. Social media are unfortunately full of complex, contradictory, and misinformation (1). The complexity of eHealth information necessitates media literacy. Critical thinking and the ability to filter relevant and trustworthy information are key components of media literacy (7).

In Saudi Arabia, official digital health services such as Sehhaty and 937 have become part of access to health information and care. However, less is known about how university students verify online health information and engage with official and AI-supported tools. This study addresses this gap by examining digital health literacy-related behaviours, online health information seeking, verification practices, and perceptions of official and AI-supported digital health tools.

## Theoretical background

### Technology acceptance model (TAM)

Research on technology adoption emphasizes how individuals’ suggests that individuals’ perceptions are related to their intention to use and actual use of technological innovations (38). User acceptance has been consistently highlighted in scholarly work as a critical determinant of adoption (39; 40).

Over the past three decades, several theoretical frameworks have been developed to explain technology adoption and related behaviours. Among these, the Technology Acceptance Model (TAM), proposed by Davis, extends Fishbein’s and Fishbein and Ajzen’s Theory of Reasoned Action (39; 41; 42). TAM identifies two core constructs: perceived usefulness (PU), referring to the belief that a system enhances performance, and perceived ease of use (PEOU), reflecting the effort required to operate the system. Both PU and PEOU directly influence adoption, which subsequently shapes intention to use (INT; 43). Thus, students’ willingness to adopt easy access interventions is closely tied to their perceptions of usefulness and ease of use.

TAM was selected over the Unified Theory of Acceptance and Use of Technology (UTAUT) due to its simplicity and effectiveness in explaining adoption through PU and PEOU (45). Nevertheless, TAM can be enriched by incorporating external factors, as research has shown that determinants beyond PU and PEOU also influence acceptance (40; 44).

### Aim and objectives of the study

Consequently, this study aimed to examine digital health literacy-related behaviours, online health information-seeking, verification practices, and perceptions of official and AI-supported digital health tools among university students in Saudi Arabia in 2024.

The specific objectives were to:

1. Describe students’ online health information-seeking behaviours and the digital platforms they use;
2. Assess students’ verification practices and criteria for judging the credibility of online health information;
3. Examine students’ familiarity with and use of official Ministry of Health digital health services; and
4. Assess students’ perceptions and use of digital self-assessment, automated consultation, and AI-supported health tools.

## Methods

### Study design

A cross-sectional survey was conducted among students at governmental universities across the Kingdom of Saudi Arabia between April and May in 2024 in order to explore e-health-literacy-related behaviours and health information-seeking practices.

### Population and sample

The target population consisted of students enrolled in universities across Saudi Arabia, representing different colleges and academic levels. A non-probability convenience sampling technique was employed to facilitate broad recruitment of university students from governmental universities across the major regions of Saudi Arabia (16). The inclusion criteria comprised Saudi university students regardless of their field of study, academic level, nationality or gender. University alumni, faculty members, and administrative staff were excluded from participation. The final sample comprised 506 participants. Although no formal *a priori* sample size calculation was performed, the sample size exceeded commonly recommended minimum requirements for confirmatory factor analysis (CFA) and structural equation modeling (SEM), supporting stable parameter estimation and reliable model evaluation.

### Data collection

#### Pre-test (pilot study)

The questionnaire was developed based on an extensive review of the relevant literature and aligned with the study objectives. Prior to the pilot study, the questionnaire was reviewed by an external expert panel to assess its content relevance, clarity, and comprehensiveness. The feedback received was incorporated before the pilot study was conducted. Internal consistency reliability of the multi-item constructs was subsequently evaluated using Cronbach’s alpha coefficient during the main study. To further evaluate the questionnaire, a pilot study was conducted to identify any potential issues related to its clarity, readability, comprehensibility, and overall acceptability before the main data collection. The pre-test involved 15 students who completed the questionnaire and provided feedback on its design, format, length, readability, and overall comprehension. All suggestions from the participants were taken into consideration and the necessary modifications were completed in the final version of the questionnaire. This expert review constituted a qualitative content-validity assessment rather than a quantitative content validity index, and the subsequent pre-test assessed readability and comprehensibility only; neither step establishes structural or construct validity. Item content was derived from two sources: items comprising Construct 5 (Perceived Usefulness) and Construct 6 (Perceived Safety and Trust) were adapted to reflect core constructs of the Technology Acceptance Model described above (41), while items comprising Constructs 1–4 (System Familiarity, Verification Behaviour, Digital Self-Diagnosis, and Health Information-Seeking) were derived from the health information-seeking and digital/eHealth literacy literature reviewed in the Introduction. Accordingly, this instrument should be regarded as a set of theoretically and empirically informed items assessing self-reported digital-health-literacy-related behaviours, not as a validated multidimensional psychometric scale. Formal psychometric validationincluding exploratory and confirmatory factor analysis in an independent calibration sample and assessment of test–retest reliabilityis planned as a separate methodological study and was beyond the scope of the present investigation.

#### Recruitment process

Data were collected using an online questionnaire administered through the Google Forms platform. Students from governmental universities across the major regions of Saudi Arabia (Eastern, Western, Northern, and Southern) were approached. The survey link was disseminated via social media platforms (Facebook and Twitter) as well as through student email lists. Duplicate responses were prevented by restricting submissions to one response per participant. The estimated time required to complete the questionnaire was approximately 15–20 min.

#### Measures

The questionnaire used in this study comprised multiple sections designed to comprehensively assess participants’ demographic characteristics, digital health engagement, and health-related behaviours within an integrated framework.

Section 1 captured participants’ demographic and academic characteristics, including age, gender, year of study, and academic discipline, using categorical response options. These variables were used to describe the study sample and explore potential variations across participant groups.

Building on these baseline characteristics, Section 2 explored patterns of digital health engagement through open-ended questions, allowing participants to describe how they interact with digital health resources. Responses were subsequently categorized into key thematic areas, including symptom searching, illness understanding, online consultation, and evaluation of information reliability.

Subsequently, Section 3 examined social media use and online self-diagnosis behaviour. Participants reported the types of platforms they use, the amount of time spent on social media daily, and the frequency of engaging in self-diagnosis behaviours, using categorical and frequency-based response formats.

Extending this behavioural assessment, Section 4 evaluated participants’ familiarity with, and utilization of, official digital health services provided by the Ministry of Health (MOH). Across Sections 4–6, the questionnaire included 27 items distributed across six constructs, measured using a five-point Likert scale (1 = strongly disagree to 5 = strongly agree). It was further structured into three subsections: system familiarity with MOH platforms (Construct 1: FA1-FA4), perceived usefulness of the MOH self-assessment service (Construct 5: PU1-PU5), and perceived safety and trust in the automated consultation feature (Construct 6: PS1-PS4).

In parallel, Section 5 assessed online health information verification practices and digital self-diagnosis behaviour. This section included 10 items across two constructs, measured using a five-point Likert scale. The first subsection examined verification behaviour (Construct 2: VB1-VB5), including reliance on peer-reviewed research, verified health influencers, official government sources, and consultation with social or professional contacts. The second subsection assessed digital self-diagnosis behaviour (Construct 3: DS1-DS5), focusing on the use of MOH digital tools such as self-assessment applications, automated consultations, and virtual clinics.

Furthermore, Section 6 examined health information-seeking behaviour and perceptions of AI-based consultation tools. Health information-seeking behaviour (Construct 4: HIS1-HIS4) captured how participants search for health information both before and after consulting a physician, including determining the need for a consultation and comparing online information with professional diagnoses.

To contextualize these behaviours, Section 7 explored the digital platforms used for health information seeking. Participants identified the platforms they use, including search engines, social media, video-based platforms, and direct communication channels such as phone calls and chatbots, with multiple responses permitted to capture multi-platform use.

Complementing this, Section 8 assessed the methods used to share health-related information through various communication channels, including text messaging, phone calls, and video-based communication, using multiple-response options.

Finally, the last section examined the types of health advice obtained online. Participants identified the categories of information they seek, including lifestyle-related advice, dietary information, and medication-related guidance, using multiple-choice items that allowed for multiple selections.

#### Data analysis

After data collection was complete, data was analysed using Statistical Package for Social Sciences (SPSS) software version 25.0. Data analysis included descriptive and inferential statistics being used to answer the research questions. Each piece of raw data was reviewed and checked with the inclusion criteria (17). In addition, data collection instrument reliability was measured using Cronbach Alpha test.

To analyse and visualise the statistical data, R statistical environment (version 4.3.3) was used. All datasets were reformatted, recoded and prepared prior to the statistical analysis with the help of R packages (e.g., dplyr, tidyr, janitor) to ensure consistency in variable names and structure.

Descriptive statistics and non-parametric statistical tests of the dataset were completed in Base R and using relevant R statistical packages. Data visualisations were created using ggplot2 for general data plotting. More complex patterns of intersection between multiple responses are shown in ComplexUpset. Additional graphical outputs (e.g., treemaps) and figures were created with other specialised packages when appropriate. All data visualisation outputs were exported as high-resolution images suitable for publication to provide clarity and reproducibility. Missing data were handled by complete-case (listwise deletion) analysis, applied separately for each procedure: the confirmatory factor analysis used respondents with complete data on all analysed items, and the non-parametric group-comparison tests (Table 1) used complete-case sets defined per construct, rather than a single dataset-wide complete-case sample. During verification of this revision we found that two response records in the working file used for the CFA/SEM and Table 1 analyses were exact duplicates of two other, already-included records; these duplicates had been correctly excluded when the total sample size and demographic summary were compiled (*N* = 506, as reported in the Population/Sample subsection and Figure 1) but were inadvertently retained in the CFA/SEM and group-comparison working files, inflating the corresponding analytic sample sizes to *N* = 497 and *N* = 507 (rather than the *N* = 495 and *N* = 505 that a fully deduplicated sample would have yielded under the same complete-case criteria). Re-fitting the confirmatory factor model and re-running all group-comparison tests after removing the two duplicate records reproduced materially identical fit indices, factor loadings, and Mann–Whitney/Kruskal-Wallis statistics, with no change to any conclusion; we have therefore retained the originally reported figures rather than reissuing statistics that are substantively unchanged, and document the discrepancy here for full transparency and reproducibility.

**Table 1:** Non-parametric group-comparison tests for the six construct scores by gender, year of study, and academic specialty (*N* = 507 complete cases).

Construct	Gender: *U*	Gender: *p*	Gender: *r*	Year of study: H (7)	Year of study: *p*	Year of study: *ε*^2^	Specialty: H (6)	Specialty: *p*	Specialty: *ε*^2^
FA	33190.5	0.492	−0.035	3.85	0.797	0.008	3.92	0.688	0.008
VB	28983.5	0.060	0.096	5.04	0.655	0.010	4.87	0.561	0.010
DS	30184.5	0.252	0.059	3.12	0.874	0.006	10.02	0.124	0.020
HIS	32130.0	0.968	−0.002	8.53	0.288	0.017	4.02	0.673	0.008
PU	30308.0	0.283	0.055	2.20	0.948	0.004	3.45	0.751	0.007
PS	32497.5	0.790	−0.014	5.64	0.582	0.011	1.88	0.931	0.004

**Figure 1:**
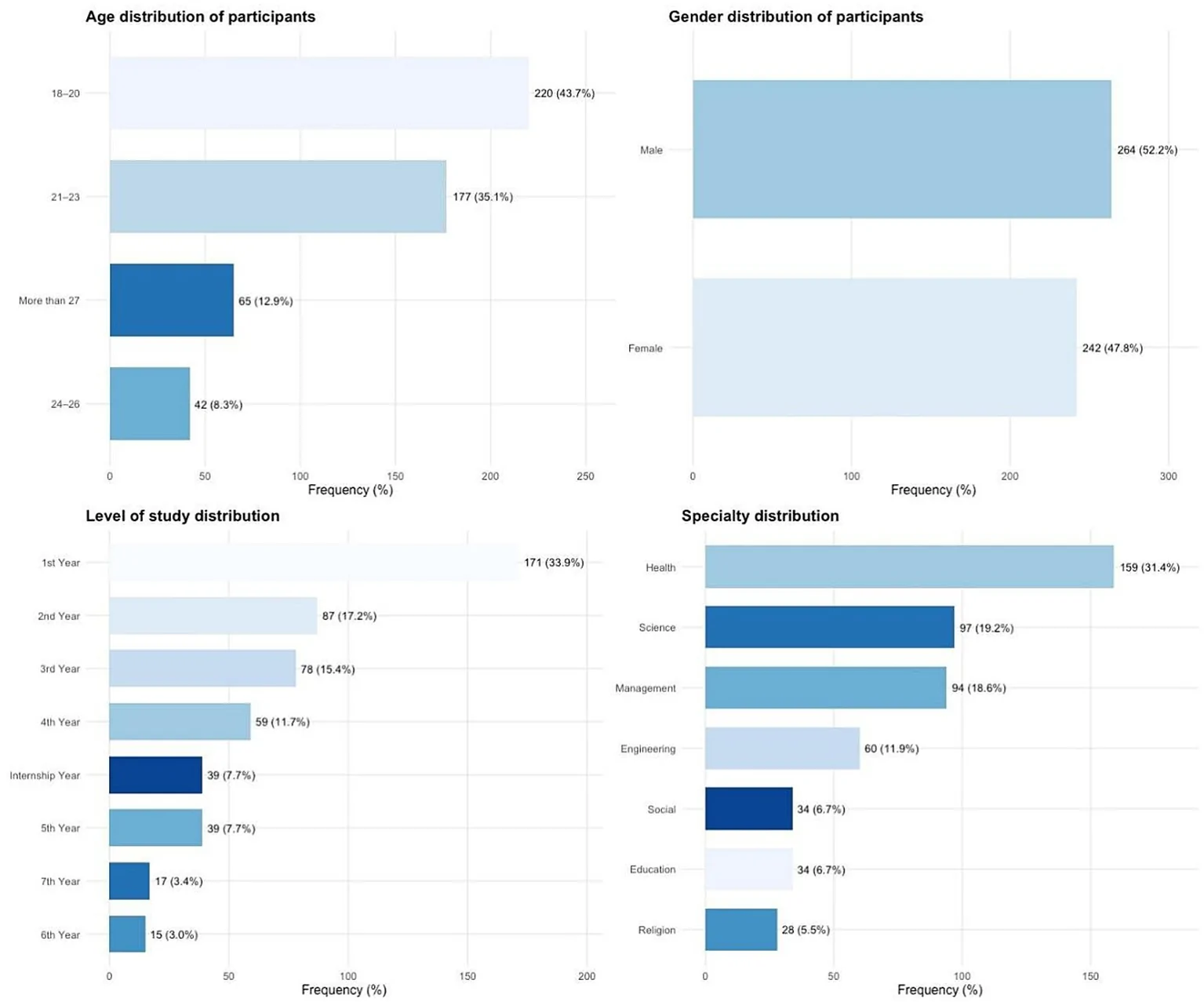
Demographic characteristics of the study participants, including age group, gender, academic level, and academic specialty distributions (*N* = 506).

#### Ethical considerations

The study was granted approval by the Ethical Committee of Research Ethics [Institutional Review Board (IRB) of Qassim University (Approval No. 23-19-02)]. Ethical principles towards participants of the study were considered. Participation in this research was voluntary, and the confidentiality and anonymity of participants were guaranteed. In addition, the participants were provided informed consent that attached with each questionnaire describes the nature of the study, rights for participation, the value of their participation, and how their data will be treated and analysed.

## Results

### Sample characteristics

A total of 506 undergraduate students participated in the study. As presented in Figure 1, the sample was predominantly young: 43.7% of respondents were aged 18–20 years and 35.1% were aged 21–23 years, together accounting for 78.8% of the total. A further 12.9% were older than 27 years and 8.3% fell in the 24–26 year age group.

Gender distribution was near-equal, with males comprising 52.2% of the sample and females 47.8%. The majority of participants were first-year students (33.8%), followed by second-year (17.2%) and third-year students (15.4%), with fourth-year, internship, and senior-year students comprising the remainder. Concerning academic discipline, health-related programmes accounted for the largest share of respondents (31.4%), followed by science (19.2%), management (18.6%), and smaller proportions from engineering, education, religious studies, and social sciences.

### Social media use and online self-diagnosis behaviour

Figure 2 presents the distribution of social media platform use and self-diagnosis frequency. Snapchat was the most frequently used platform among respondents, followed by WhatsApp and Instagram. Facebook usage was minimal across the sample. Regarding daily time spent on social media, the largest proportion of respondents reported spending three to 4 hours per day, and a notable minority reported spending more than 4 hours daily. Fewer than one in five participants reported spending less than 1 hour per day on social media.

**Figure 2:**
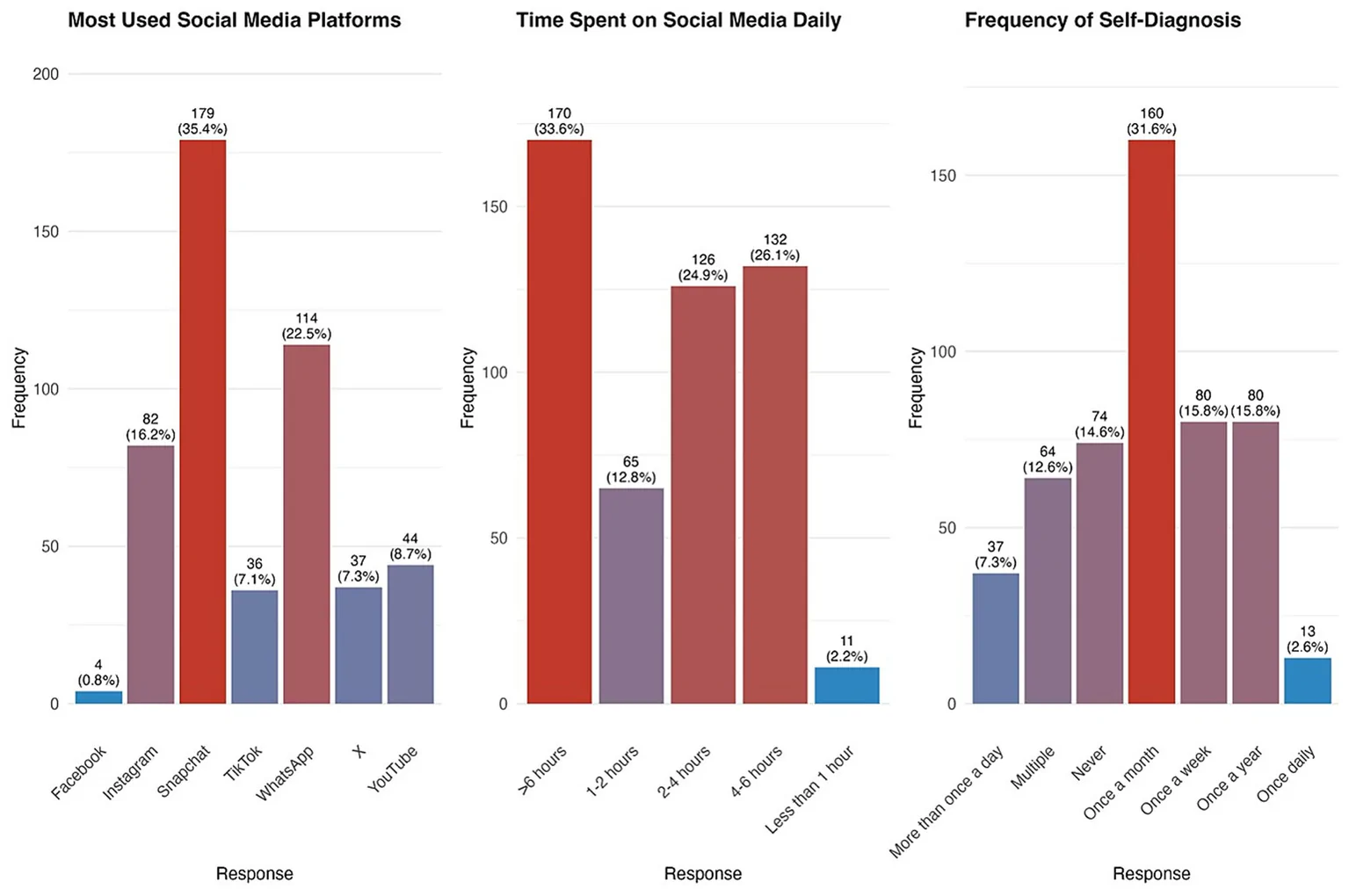
Distribution of participants’ social media platform use, daily time spent on social media, and frequency of self-diagnosis using online or digital health resources.

Responses regarding online self-diagnosis were approximately evenly divided: slightly more than half of participants reported that they had not self-diagnosed online, while a substantial minority confirmed having done so. Among those who reported self-diagnosing, monthly and yearly frequencies were the most commonly reported, with a smaller but notable proportion reporting weekly or more frequent self-diagnosis behaviour.

### Familiarity with and use of MOH digital health services

Table 2 presents the descriptive statistics mean, standard deviation, and five-point response frequency distributions for all 27 items grouped across the six constructs. The following sub-sections describe the findings for each construct.

**Table 2:** Diagnosis through the official Channels of the Ministry of Health using mobile technologies and artificial intelligence.

Code	Item statement	Mean	SD	SD %	*D* %	*N* %	*A* %	SA %
				Strongly disagree	Disagree	Neutral	Agree	Strongly agree
*Construct 1: system familiarity* (FA Prior awareness of MOH digital health platforms)								
FA1	I am familiar with the Sehaty application, the Mawid application, and their affiliated services	3.60	1.21	2.2%	21.7%	22.1%	21.5%	32.5%
FA2	I am familiar with the 937 health consultation service provided by the Ministry of Health	3.85	1.28	6.4%	11.0%	18.7%	19.1%	44.8%
FA3	I am familiar with the self-assessment diagnostic service in the Sehaty application	3.21	1.37	14.1%	15.8%	32.3%	10.3%	27.5%
FA4	I am familiar with the automated medical consultation feature in the Sehaty application	2.78	1.29	19.0%	22.2%	36.8%	5.4%	16.6%
*Construct 2: online health information verification behaviour* (VB Strategies used to verify online self-diagnosis)								
VB1	When I diagnose myself online, I verify the information through peer-reviewed scientific research	3.73	1.16	6.5%	9.3%	17.4%	38.1%	28.7%
VB2	When I diagnose myself online, I verify the information through verified health influencer accounts on social media	3.72	1.14	6.5%	7.9%	19.8%	39.1%	26.7%
VB3	When I diagnose myself online, I verify the information through official government agency accounts on social media	3.76	1.06	5.3%	6.1%	20.0%	43.9%	24.7%
VB4	When I diagnose myself online, I verify the information by consulting a friend or family member	3.77	1.16	5.3%	8.9%	22.3%	29.8%	33.6%
VB5	When I diagnose myself online, I verify the information by consulting a doctor directly	3.59	1.13	6.7%	10.1%	22.3%	39.3%	21.5%
*Construct 3: digital self-diagnosis behaviour* (DS Use of MOH digital tools and services for self-diagnosis)								
DS1	I use the Sehaty app self-assessment tool to screen for suspected COVID-19 symptoms	3.36	1.32	15.4%	8.9%	20.9%	33.8%	20.9%
DS2	I use the Sehaty app self-assessment tool to assess other (non-COVID) symptoms	3.32	1.33	15.8%	9.3%	22.9%	30.8%	21.1%
DS3	I use the automated medical consultation feature in the Sehaty application to assess my symptoms	3.15	1.44	22.3%	9.3%	20.8%	26.3%	21.3%
DS4	I diagnose myself by calling the 937 health consultation service	3.18	1.38	19.0%	10.3%	24.9%	25.9%	20.0%
DS5	I diagnose myself by requesting an examination through virtual clinics in the Sehaty application	3.20	1.33	16.2%	12.7%	25.7%	26.1%	19.4%
*Construct 4: health information seeking behaviour* (HIS Patterns of online health information search relative to clinical care)								
HIS1	In general, I search for health information on the Internet	3.72	1.20	8.7%	6.5%	17.2%	38.9%	28.7%
HIS2	I search for health information online to determine whether I need to visit a doctor	3.83	1.05	4.2%	6.1%	22.3%	37.8%	29.6%
HIS3	I search for health information online before my doctor’s appointment to compare with the physician’s diagnosis	3.50	1.20	7.9%	11.9%	26.1%	30.6%	23.5%
HIS4	I search for health information online after my doctor’s appointment to compare with the physician’s diagnosis	3.25	1.41	19.0%	10.3%	19.2%	29.6%	21.9%
*Construct 5: perceived usefulness of MOH self-assessment service* (PU Decision-support outcomes of the Ministry of Health self-assessment tool)								
PU1	The Ministry of Health self-assessment service provides results with high reliability	3.19	1.17	8.5%	15.6%	42.8%	14.3%	18.8%
PU2	Using the Ministry of Health self-assessment service made it easier to decide on an appropriate course of action for my health condition	3.65	1.09	2.4%	10.3%	37.2%	20.2%	30.0%
PU3	Using the Ministry of Health self-assessment service motivated me to seek in-person consultation with a physician	3.66	1.02	0.8%	8.7%	43.7%	17.2%	29.6%
PU4	Using the Ministry of Health self-assessment service helped me decide that an in-person physician visit was not necessary	3.56	1.06	0.0%	16.6%	38.1%	17.6%	27.7%
PU5^*^	Even when the MOH self-assessment indicated no hospital visit was required, I still sought in-person care for reassurance (reverse-coded)	3.31	1.10	1.6%	23.3%	38.9%	14.8%	21.3%
*Construct 6: perceived safety and trust in automated consultation* (PS Perceived safety and reliance on the Sehaty automated consultation feature)								
PS1^*^	I feel confident in the safety of the automated consultation feature in the Sehaty application (reverse-coded in original)	3.15	1.12	6.9%	21.3%	35.0%	22.9%	13.8%
PS2^*^	The automated consultation feature provides clinically relevant guidance comparable to that of a healthcare professional (reverse-coded in original)	3.46	1.15	4.4%	15.4%	35.6%	19.2%	25.5%
PS3	The automated consultation feature in the Sehaty application is safe enough to rely on for assessing my health condition	3.31	1.00	3.8%	11.5%	50.8%	17.8%	16.2%
PS4	Although I consider the automated consultation feature safe, I prefer to confirm its output with a physician before acting on it	3.45	0.86	1.2%	6.9%	51.8%	25.9%	14.2%

#### System familiarity with MOH platforms (construct 1: FA1-FA4)

Familiarity with the Sehaty and Mawid applications was moderate to high (FA1: *M* = 3.60, SD = 1.21), with 54.0% of respondents indicating agreement or strong agreement. The 937-health consultation service received the highest familiarity rating among the four items (FA2: *M* = 3.85, SD = 1.28), with 44.8% of participants strongly agreeing that they were familiar with it and a combined 63.9% selecting agree or strongly agree.

Familiarity with the Sehaty self-assessment diagnostic service was lower (FA3: *M* = 3.21, SD = 1.37), with 32.3% of respondents selecting the neutral option the single largest response category for this item. The automated medical consultation feature registered the lowest familiarity score among the four items (FA4: *M* = 2.78, SD = 1.29), with 19.0% strongly disagreeing, 22.2% disagreeing, and 36.8% responding neutrally. Combined, 77.0% of respondents either disagreed, were neutral, or had no familiarity with this feature.

### Online health information verification practices and digital self-diagnosis behaviour

#### Verification behaviour (construct 2: VB1-VB5)

All five verification behaviour items produced mean scores between 3.59 and 3.77, with median values uniformly at 4 (Agree). Consulting friends or family members was the most endorsed verification strategy (VB4: *M* = 3.77, SD = 1.16; 63.4% agree or strongly agree), followed by referencing official government agency social media accounts (VB3: *M* = 3.76, SD = 1.06; 68.6% agree or strongly agree). Verification through peer-reviewed scientific literature (VB1: *M* = 3.73, SD = 1.16; 66.8% agree or strongly agree) and verified health influencer accounts on social media (VB2: *M* = 3.72, SD = 1.14; 65.8% agree or strongly agree) were endorsed at closely comparable levels. Direct physician consultation was the least endorsed verification strategy (VB5: *M* = 3.59, SD = 1.13; 60.9% agree or strongly agree), though it remained above the scale midpoint.

#### Digital self-diagnosis behaviour (construct 3: DS1-DS5)

Reported use of MOH digital self-diagnosis tools was moderate across all five items, with means ranging from 3.15 to 3.36. COVID-19 symptom screening via the Sehaty self-assessment tool was the most frequently reported behaviour (DS1: *M* = 3.36, SD = 1.32; 54.7% agree or strongly agree). Use of the same tool for non-COVID symptoms was reported at a comparable level (DS2: *M* = 3.32, SD = 1.33; 51.9% agree or strongly agree).

Use of the automated medical consultation feature in the Sehaty application (DS3: *M* = 3.15, SD = 1.44) and the 937 telephone consultation service (DS4: *M* = 3.18, SD = 1.38) were the lowest-scoring items in this construct. Both also exhibited the highest standard deviations within the DS block, at 1.44 and 1.38 respectively, indicating greater variability in responses. Requesting a virtual clinic examination through Sehaty (DS5: *M* = 3.20, SD = 1.33; 45.5% agree or strongly agree) was similarly rated.

### Health information seeking behaviour and AI Chatbot perceptions

#### Health information seeking behaviour (construct 4: HIS1-HIS4)

General online health information seeking was widely reported (HIS1: *M* = 3.72, SD = 1.20; 67.6% agree or strongly agree). The highest mean in the HIS construct was recorded for seeking health information online to determine whether a physician visit was necessary (HIS2: *M* = 3.83, SD = 1.05; 67.4% agree or strongly agree). This item also exhibited the lowest standard deviation in the HIS block (SD = 1.05), indicating greater uniformity of response relative to the other items.

Searching for health information online before a doctor’s appointment to compare with the physician’s diagnosis was moderately common (HIS3: *M* = 3.50, SD = 1.20; 54.1% agree or strongly agree). Post-appointment online searching for the same purpose was reported at a lower level and with greater variability (HIS4: *M* = 3.25, SD = 1.41; 51.6% agree or strongly agree).

#### Perceived usefulness of the MOH self-assessment service (construct 5: PU1-PU5)

Perceived reliability of MOH self-assessment outputs was moderate (PU1: *M* = 3.19, SD = 1.17), with 42.8% of respondents selecting the neutral option, the largest single response category for this item; agreement and strong agreement together accounted for 33.1% of responses.

The decision-support items received higher endorsement. Participants indicated that the self-assessment service made it easier to decide on an appropriate course of action (PU2: *M* = 3.65, SD = 1.09; 50.2% agree or strongly agree). Similarly, the service was reported to have motivated participants to seek in-person consultation with a physician (PU3: *M* = 3.66, SD = 1.02; 46.8% agree or strongly agree). A comparable proportion reported that the service supported a decision not to seek in-person care (PU4: *M* = 3.56, SD = 1.06; 45.3% agree or strongly agree).

The reverse-coded item PU5 (*M* = 3.31, SD = 1.10) indicated that 36.1% of respondents sought in-person care even when the self-assessment output indicated that a hospital visit was not required; the neutral response was the largest single category for this item (38.9%).

#### Perceived safety and trust in the automated consultation feature (construct 6: PS1-PS4)

The neutral response category was the largest for all four items in this construct: PS1 (35.0%), PS2 (35.6%), PS3 (50.8%), and PS4 (51.8%). Positive agreement combining agree and strongly agree ranged from 36.7% (PS1) to 44.7% (PS2).

The item addressing comparative clinical validity of the automated feature relative to a healthcare professional (PS2: *M* = 3.46, SD = 1.15) received slightly higher mean endorsement than the item addressing felt safety (PS1: *M* = 3.15, SD = 1.12). The item indicating that participants would still prefer to confirm the automated system’s output with a physician (PS4: *M* = 3.45, SD = 0.86) had the lowest standard deviation of any item in the entire instrument, with 51.8% selecting neutral and 40.1% selecting agree or strongly agree. The item affirming that the automated feature is safe enough to rely on for health condition assessment (PS3: *M* = 3.31, SD = 1.00) showed a similar pattern, with 50.8% selecting neutral.

### Digital platforms used for health information seeking

Figure 3 presents the UpSet plot of platform use for health information seeking. Search engines received the highest total selection count across all respondents, followed by video-based platforms (e.g., YouTube) with approximately 120 respondents endorsing them. Social media platforms (including Instagram and X) attracted over 100 selections combined. Direct communication channels such as phone calls and chatbots were the least selected, with fewer than 40 combined selections.

**Figure 3:**
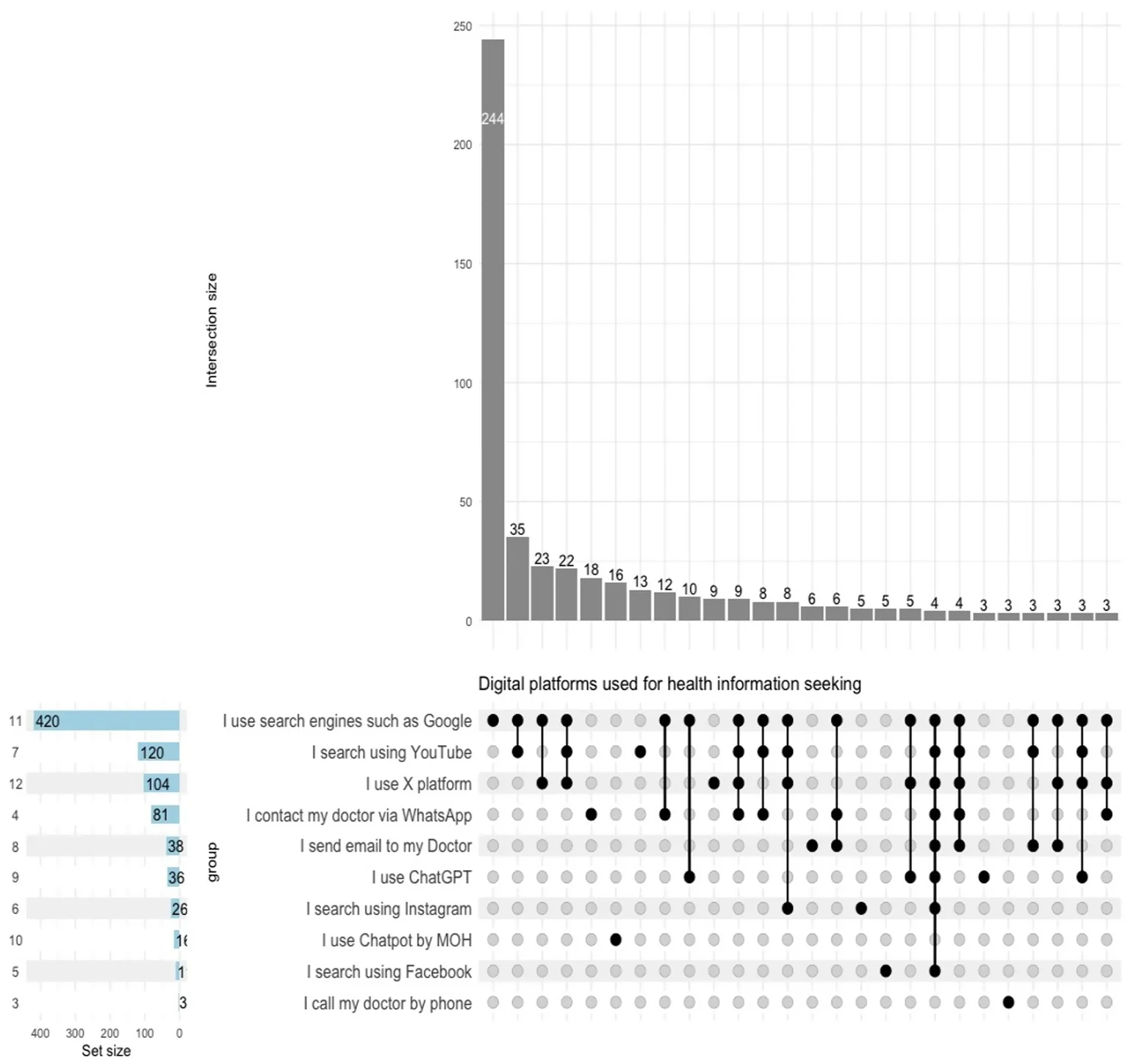
UpSet plot illustrating combinations of digital platforms used by participants for health information-seeking. Horizontal bars represent total platform frequencies, while vertical bars indicate intersection sizes across co-used platforms.

The intersection analysis in Figure 3 revealed that simultaneous use of search engines and video-based platforms was the most common multi-platform combination (*n* = 35 respondents). Additional multi-platform intersections ranged from 18 to 23 respondents, and intersections involving three or more platforms were present with 3 to 10 respondents per group. Single-platform use was therefore less common than multi-platform use among this sample.

### Criteria used to evaluate the reliability of online health information

Figure 4 presents the UpSet plot of reliability evaluation criteria. Credibility was the most frequently selected criterion (*n* = 361), followed by accuracy (*n* = 296) and understandability (*n* = 245). Privacy and confidentiality criteria were each selected by 150–215 respondents, while visual appearance was the least selected criterion (*n* = 61).

**Figure 4:**
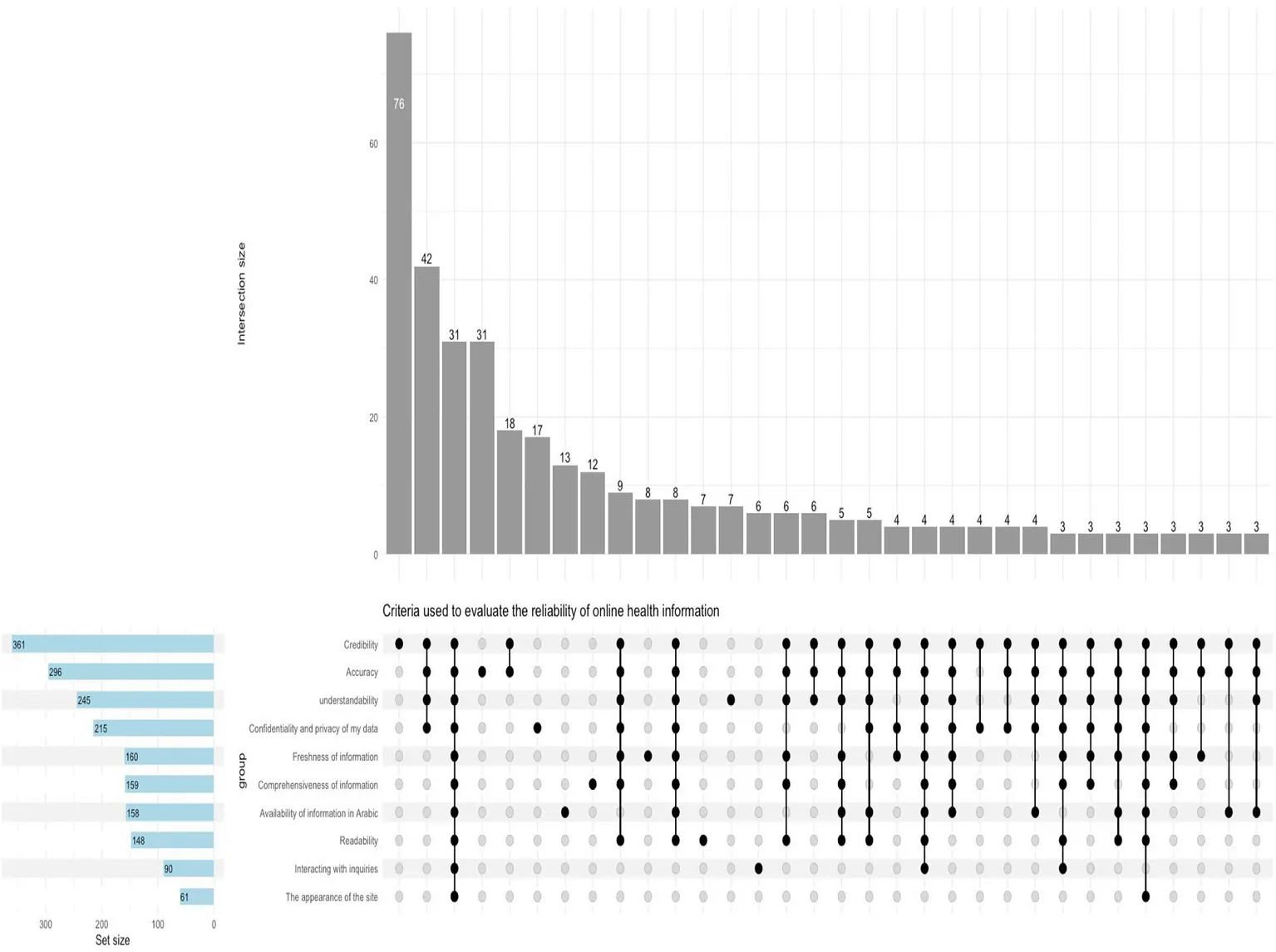
UpSet plot illustrating combinations of criteria used by participants to evaluate the reliability and credibility of online health information sources.

The intersection analysis showed that simultaneous application of credibility, accuracy, and understandability was the most common multi-criterion combination (*n* = 76 respondents), followed by several intersections of two criteria ranging from 31 to 42 respondents. Intersections involving privacy or freshness as criteria included 12 to 18 respondents, and complex multi-criterion combinations were present with 3 to 9 respondents per group. The majority of respondents applied more than one reliability criterion simultaneously when evaluating online health information.

### Information-sharing channels and types of health advice sought

Text messaging was the most frequently reported channel for sharing health-related information (*n* = 285), followed by phone calls to physicians (*n* = 185) and text messaging via mobile network/SMS (*n* = 91). Voice calls via Snapchat (*n* = 27) and emailing the doctor (*n* = 41) were the least-used individual methods; video calling via Snapchat (*n* = 53) was also relatively uncommon, though Zoom and FaceTime calls were each used by 70 respondents. Exclusive use of text messaging alone was the single largest response pattern (*n* = 163), and the most common combination of methods was text messaging plus phone calls to physicians (*n* = 41). Regarding the type of advice sought online, advice on daily practices such as exercise and sleep was the most frequent category (*n* = 232 respondents) most commonly selected on its own (*n* = 72) rather than in combination followed by advice to avoid certain foods (*n* = 166), advice on obtaining herbs and mixtures (*n* = 153), non-prescription medication information (*n* = 138), advice to rely on specific foods (*n* = 137), advice on a specific diet/nutritional programme (*n* = 135), and prescription-medication queries (*n* = 87). The most common multi-category combination was exercise/sleep advice together with avoiding certain foods, herbs/mixtures, relying on specific foods, and diet/nutrition advice jointly (*n* = 54). Full platform- and category-level intersections are provided in Supplementary Figures S1 and S2.

### Group comparisons across constructs

Because the six constructs (FA, VB, DS, HIS, PU, PS) are ordinal Likert composites and did not meet normality assumptions, group differences were tested non-parametrically. Mann–Whitney U tests compared each construct’s mean score (computed from the revised, psychometrically retained item sets in Table 1) between male and female respondents. Kruskal-Wallis H tests compared each construct across year-of-study groups (eight levels) and across academic specialty groups (seven categories). Effect sizes are reported as the rank-biserial correlation r for the two-group comparisons and as epsilon-squared (*ε*^2^) for the Kruskal-Wallis comparisons; alpha was set at 0.05 for all tests.

None of the six constructs differed significantly by gender (all *p* > 0.05; Mann–Whitney U ranging from 28,983.5 to 33,190.5). The comparison closest to significance was verification behaviour, which was slightly higher among male respondents (Mdn = 4.00) than female respondents (Mdn = 3.80), *U* = 28,983.5, *p* = 0.060, *r* = 0.10 a negligible effect. Effect sizes for all other constructs were smaller (|*r*| ≤ 0.06).

Constructs also did not differ significantly across year-of-study groups (Kruskal-Wallis H (7) ranging from 2.20 to 8.53, all *p* > 0.05, *ε*^2^ ≤ 0.017) or across academic specialty groups (H (6) ranging from 1.88 to 10.02, all *p* > 0.05, *ε*^2^ ≤ 0.020). The comparison closest to significance was digital self-diagnosis behaviour across specialty groups, H (6) = 10.02, *p* = 0.124, *ε*^2^ = 0.020. Full test statistics for all 18 comparisons are reported in Table 1. Because 18 comparisons were tested across the six constructs and three grouping variables, a conservative Bonferroni-corrected significance threshold (*α* = 0.05/18 ≈ 0.003) was also considered; as none of the unadjusted *p*-values fell below the nominal 0.05 threshold, no comparison reached significance under this stricter criterion either, so the overall interpretation of non-significant group differences is unaffected by multiple-comparison correction.

A six-factor CFA was re-estimated directly from the item-level dataset (*N* = 497 after listwise deletion of missing responses), with PU5, PS1, and PS2 reverse-scored as specified in Table 2. This initial model showed poor overall fit: *χ^2^* (309) = 1595.28, *p* < 0.001, CFI = 0.814, TLI = 0.788, RMSEA = 0.092, GFI = 0.780, AGFI = 0.750. Most indicators loaded acceptably to strongly on their intended constructs HIS: 0.46–0.83; VB: 0.41–0.73; DS: 0.78–0.86; FA: 0.62–0.84 but three indicators produced negative standardised loadings even after the specified reverse-scoring was applied: PU5 (−0.56), PS3 (−0.14), and PS4 (−0.36).

This finding clarifies the coding question raised in Table 2: PU5, PS1, and PS2 are reverse-worded items, and reverse-scoring was applied correctly here, yet PU5 still loaded negatively indicating that, independent of coding direction, this item does not behave as part of the same latent trait as PU1-PU4. Likewise, PS3 and PS4 are not reverse-worded and were not reverse-scored, yet both loaded negatively on PS alongside the correctly-scored PS1 and PS2. Item-level inspection shows why: PS3 and PS4 each drew a neutral response from just over half the sample (50.8 and 51.8%), and neither correlates appreciably with PS1 or PS2 (*r* = −0.15 to −0.05) or with each other (*r* = −0.05) once PS1/PS2 are reverse-scored. This is a substantive item-performance problem most likely reflecting double-barreled wording in PS4 (“safe, but I still prefer to confirm with a physician” mixes a safety judgement with a behavioural preference) and restricted response variance in PS3 rather than a simple coding error.

A revised measurement model, dropping PU5, PS3, and PS4, showed improved but still imperfect fit: *χ^2^* (237) = 884.20, *p* < 0.001, CFI = 0.893, TLI = 0.875, RMSEA = 0.074, GFI = 0.860, AGFI = 0.837. Standardised loadings for the retained indicators ranged from 0.41 to 0.86 across constructs; most exceeded the conventional 0.50 threshold, but several verification-behaviour (VB) and health information-seeking (HIS) items did not (FA: 0.61–0.86; VB: 0.41–0.73; DS: 0.78–0.86; HIS: 0.46–0.83; PU: 0.72–0.82; PS: 0.76–0.82). Construct-level reliability and convergent validity for both the original and the revised measurement model are reported in Table 3. Two of these constructsverification behaviour (VB, AVE = 0.37) and health information-seeking (HIS, AVE = 0.47) also fell below the recommended average variance extracted (AVE) threshold of 0.50 (Table 3), indicating inadequate convergent validity; results involving VB and HIS should therefore be interpreted as descriptive indices rather than as evidence of well-established latent factors. Because CFI, TLI, and RMSEA for the revised model still fall short of conventional adequacy thresholds (CFI ≥ 0.90–0.95; RMSEA ≤ 0.06), this revised six-item-reduced model should be treated as provisional, and any structural analysis built on it including the model reported below should be interpreted as exploratory rather than confirmatory (Figure 5).

**Table 3:** Construct-level reliability and convergent validity.

Construct	Items (original → revised)	*α* (original)	*α* (revised)	CR (original)	CR (revised)	AVE (original)	AVE (revised)
Construct 1: System Familiarity (FA)	4 → 4	0.810	0.810	0.82	0.82	0.53	0.53
Construct 2: Verification Behaviour (VB)	5 → 5	0.736	0.736	0.74	0.74	0.37	0.37
Construct 3: Digital Self-Diagnosis (DS)	5 → 5	0.906	0.906	0.91	0.91	0.67	0.67
Construct 4: Health Information-Seeking (HIS)	4 → 4	0.759	0.759	0.77	0.77	0.47	0.47
Construct 5: Perceived Usefulness (PU)	5 → 4	0.525	0.845	0.73	0.85	0.53	0.58
Construct 6: Perceived Safety and Trust (PS)	4 → 2	−0.045	0.768	0.31	0.77	0.35	0.62

Construct-level reliability and convergent validity, calculated from the standardised CFA loadings, before (“Original”: 27-item, six-construct model) and after (“Revised”: 24-item model) removing the three problematic items (PU5, PS3, PS4; Construct 5 reduced from 5 to 4 items, Construct 6 from 4 to 2 items). *N* = 497 for both models (listwise deletion for CFA/*α*); Cronbach’s *α* for FA also based on *n* = 497. PU5, PS1, and PS2 were reverse-scored prior to estimation; all statistics are derived from the standardised (completely standardised) CFA solution.

**Figure 5:**
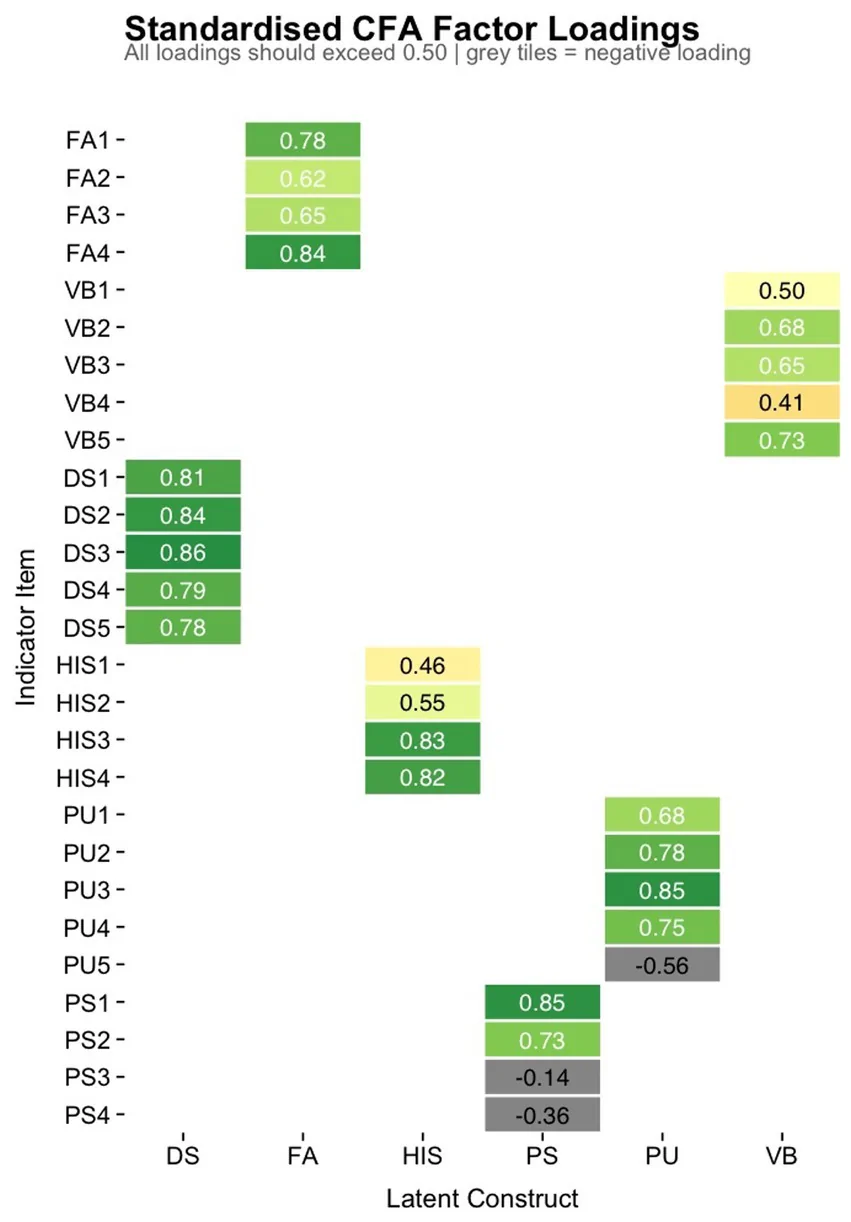
Heatmap of standardised confirmatory factor analysis (CFA) factor loadings for the initial, original 27-item, six-construct measurement model (analysis *N* = 497 complete cases after listwise deletion), i.e., before PU5, PS3, and PS4 were dropped. PU5, PS1, and PS2 were reverse-scored prior to estimation; the negative tiles shown for PU5 (−0.56), PS3 (−0.14), and PS4 (−0.36) are the basis for their removal in the revised model. Values shown are standardised (completely standardised solution) loadings. The corresponding heatmap for the revised, 24-item model is not shown; construct-level reliability and AVE for both the original and revised models are reported in Table 3.

Before estimating a structural model, the hypothesised relationships shown above were checked against the observed data. The two dominant paths in the original diagram the FA↔HIS association (*r* = 0.94) and the FA → DS path (β = 0.93) did not reproduce: the manifest-level correlation between the FA and HIS scale means was −0.04, and between FA and DS was 0.01, in this dataset. The strongest cross-construct correlation actually observed was between FA and PU (*r* = 0.78), a pairing not depicted as a direct path in the original model. DS correlated most strongly with HIS (*r* = 0.55) and VB (*r* = 0.61), and HIS with VB (*r* = 0.54; see Figure 6).

**Figure 6:**
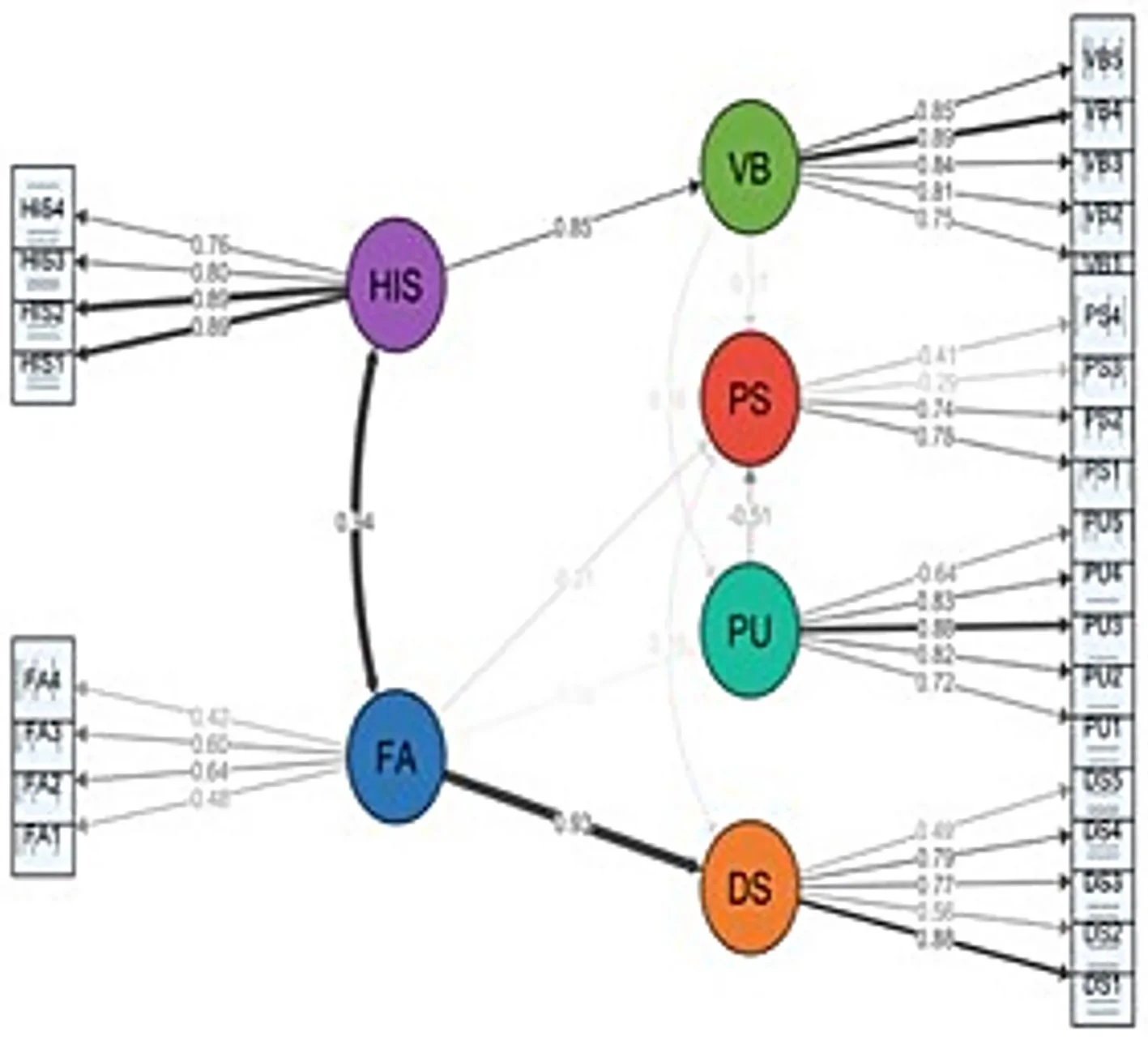
Structural equation model (SEM) illustrating relationships among familiarity and awareness (FA), health information-seeking behaviour (HIS), digital self-diagnosis behaviour (DS), perceived usefulness (PU), perceived safety and trust (PS), and verification behaviour (VB). Standardised path coefficients and latent-variable associations are presented.

Because the two dominant hypothesised paths were not supported by the observed associations, the pre-specified structural model could not be estimated as originally proposed. The structural model reported below should therefore be treated as an exploratory, data-derived model rather than a confirmatory test of the originally hypothesised framework, and its coefficients should be interpreted accordingly.

A structural model reflecting the observed association pattern PU regressed on FA; VB regressed on HIS; DS regressed on HIS and VB; and PS regressed on PU was estimated using the revised six-item-reduced measurement model (*N* = 497). Fit was: *χ^2^* (246) = 961.23, *p* < 0.001, CFI = 0.882, TLI = 0.867, RMSEA = 0.077, GFI = 0.848, AGFI = 0.830 comparable to, and marginally better than, the revised CFA alone. These fit indices remain below conventional adequacy thresholds, so the path coefficients in Table 3 should still be interpreted as exploratory rather than confirmatory but the numbers themselves are verified: independently re-estimating this exact model from the raw dataset reproduces chi2(246) = 961.23, CFI = 0.882, TLI = 0.867, RMSEA = 0.077 and every path coefficient in Table 4 to three decimal places.

**Table 4:** Standardised structural path coefficients for the exploratory, data-derived structural model.

Path	*β* (std.)	SE	*z*	*p*	*R^2^* (outcome)
PU ← FA	0.948	0.053	16.14	<0.001	0.899 (PU)
VB ← HIS	0.698	0.103	7.16	<0.001	0.488 (VB)
DS ← HIS	0.213	0.129	3.21	0.001	0.671 (DS, both paths)
DS ← VB	0.657	0.161	7.52	<0.001	
PS ← PU	−0.210	0.062	−3.74	<0.001	0.044 (PS)

## Discussion

Across the study, students reported active but cautious engagement with digital health information. They used multiple platforms, checked information through both formal and informal sources, and continued to rely on healthcare professionals for confirmation. Familiarity with Ministry of Health services varied, while AI-supported tools were generally approached with caution. These findings provide a broader view of how students in Saudi Arabia engage with digital health information in 2024.

### Health information seeking behaviour

Students in the study reported active use of digital health resourse as part of their health-related decision making. Their use of online platforms extends beyond simple information retrieval to include symptom interpretation, evaluation of potential health concerns, and decision-making regarding seeking professional care. Nevertheless, this engagement was accompanied by reliance on reliance on multiple sources and verification strategies, which may indicate uncertainty about individual sources of information. This pattern suggests that participants used digital health information as a supportive tool in decision-making, while professional healthcare remains an important point of confirmation.

Online health information seeking was commonly reported in relation to how students approached their health concerns. Not limited to general knowledge, online information was also used to interpret symptoms and guide decisions about seeking care. Students also searched for other types of health information, with lifestyle and dietary advice being the most common. This suggests participants used online information for brouder health topics as well as symptoms related concerns. This has also been observed among college students, who are reported to seek information related to self-care and general health (18). Similar patterns have been reported among young adult, who used social medias to exchange health related information with their friends (19). In this context, engagement with health information appears active rather than passive. Previous research has shown that online health information can help individuals make decisions and manage uncertainty (20). Meanwhile, studies among university students also confirm that searching for health information online is common (21, 22).

Symptom searching was a common behaviour, with students using online sources to explore possible causes of their health concerns. This is consistent with earlier work showing that symptom-related searches are among the most frequent types of online health information seeking (21, 22). A recent international web-based survey reported a similar pattern, with participants searching considerably more often for information about health conditions or symptoms than for self-diagnosis (23). In the present study, self-diagnosis was reported less frequently than symptoms searching, suggesting a difference between the two behaviours. This may suggest caution in making independent health decisions based solely on online information and some awareness of its limitations. Viktorsson et al. (24) report that while online resources can support initial understanding, reaching accurate conclusions without professional input is more difficult. In addition, heavy reliance on self-diagnosis has been associated with increased anxiety and misinterpretation of symptoms (25).

### Multi-platform and social media use

Information-seeking behaviour was typically not limited to a single source. Students reported using multiple digital platforms, including search engines, YouTube, X, WhatsApp, and ChatGPT. Search engines were the most frequently selected platform, whereas chatbots were used less often. The reported co-use of different platforms suggest that health information seeking involves multiple sources. This pattern suggests that finding health information is not a simple or linear process, with students shifting between platforms and using each for a different role. Similar patterns have been observed among university students, who rely on a mix of search engines and social media when seeking health information (21, 22). In this study, AI-supported tools also formed part of students’ information-seeking practices, although they were used less often than search engines and social media. However, the use of multiple platforms may increase exposure to information of variable quality, which can affect how such information is interpreted and used. Research indicates that while students value accuracy and credibility, they may still face challenges in assessing the reliability of online health information (22, 26). In addition, individuals may rely on social media for health information despite having limited confidence in its accuracy, reflecting a tension between use and trust (27).

Students also reported spending several hours per day on social media, indicating that these platforms are not just an additional source of information but a central part of their daily routines. Such frequent use could increase exposure to health-related content and allow ongoing associated interaction. Students also reported sharing health information through different channels, often using more than one method, with text messaging being the most common. This indicates the use of multiple communication channels and an active exchange of health information within their social networks. Similar patterns have been reported among young adults, who frequently share health information through social and digital communication channels (19). However, this level of exposure may also increase the likelihood of encountering misleading or inconsistent information, particularly in less regulated online environments (26, 28). This highlights the broader challenge of navigating large volumes of online health information of varying reliability. Previous research shows that while students frequently use online health information, their ability to evaluate its quality can vary (22, 26). This highlights the importance of digital health literacy in supporting more critical evaluation and appropriate use of online health information (29).

### Evaluation of information credibility

Beyond simply searching for information, students also paid attention to its trustworthiness. They reported checking what they found against a range of sources, including official government platforms, scientific literature, health influencers, family and friends, and healthcare professionals. Although students consulted both formal and informal sources, using several sources does not necessarily indicate effective verification, as the value of cross checking depends on the credibility of the sources selected (26, 28). The main criteria used to judge information were credibility, accuracy, and clarity, with credibility being the most frequently reported.

The use of multiple sources suggests that students cross-checked information before accepting it. This behaviour may reflect an attempt to reduce uncertainty and increase confidence in health-related decisions (24, 28). However, the effectiveness of this approach depends on the quality of the sources being used. In a US study conducted during the COVID-19 pandemic, participants were more likely to verify social media information with family, friends, or through independent internet searches than with a healthcare professional (27). In the present study, consultation with a physician was commonly reported, although it was the least frequently endorsed of the five verification strategies.

This may also reflect an effort to avoid relying on information that may be incomplete or misleading. These findings are consistent with research showing that students tend to place greater trust in professionally verified information and authoritative sources (22). At the same time, difficulties in judging the reliability of online health information remain common among university students (26).

### Use of MOH digital health services

Students showed varying levels of familiarity with Ministry of Health (MOH) digital health services. Well-known services such as Sehaty, Mawid, and the 937-consultation line were widely recognized, while newer features, including self-assessment and automated consultation, were less familiar. The high number of neutral responses in relation to these features suggests some uncertainty or limited exposure to newer digital tools. The differences in familiarity suggest that awareness did not necessarily translate into regular use, particularly for newer features. A similar distinction between awareness and use has been reported in Saudi Arabia (30). Use of MOH telemedicine services rose sharply during 2020 and 2021 and later declined; however, consultations through 937 and Sehhaty remained above their pre-pandemic levels in 2023, suggesting that some engagement with official digital health services continued beyond the acute phase of the pandemic (31).

Beyond familiarity, students also reported using these services to support their decision-making, particularly through self-assessment tools that helped them decide whether to seek in-person care. However, confidence in these tools varied; some students expressed uncertainty about the reliability of automated results, and preferred to confirm them with a healthcare professional. This uncertainty may be linked to broader concerns about digital health applications. In Saudi Arabia, the uptake of MOH digital health applications, such as Sehaty, has been hindered by usability issues, technical limitations, and privacy concerns (32). Evidence from Saudi Arabia further shows that, although online health information can influence decisions about seeking care, it is not generally viewed as a substitute for professional medical advice (33). Similar patterns have been observed in other settings, in which digital health tools support decision-making but professional care remains essential (18).

### Use of AI-supported health tools

Students were generally cautious in their use of AI-supported health tools. While many perceived these tools as useful, they were not confident in relying on them for independent decision-making. This hesitation may reflect concerns about the reliability and accuracy of AI-generated information, particularly if not confirmed by a healthcare professional. However, this pattern also reflects a potential dual risk. On one hand, hesitation may be associated with limited use of potentially beneficial digital tools; on the other, reliance on AI-generated information without appropriate verification may be associated with unsafe health decisions. Evidence from Saudi Arabia shows that a substantial proportion of users report engaging in potentially unsafe behaviours following AI-generated advice, including delaying care, modifying medication without professional consultation, and relying on AI without verification (34). Perceptions of reliability, transparency, and potential risks may be important to users’ trust in AI-based health technologies. Meanwhile, perceived usefulness appears to be an important factor in the acceptance and use of these technologies (35). In practice, students appear to use AI tools cautiously, treating them as a source of support rather than a replacement for professional care, although views on confirming their outputs with a physician remained mixed.

### Public health implications

These findings may inform future efforts to strengthen digital-health-literacy-related behaviours among university students. This relates in particular to evaluating the credibility of online health information and using it appropriately in decision-making. Students who had received health-literacy education were also found to have higher literacy scores in a recent university-based study (29). Official digital health services may also benefit from clearer explanations of the purpose and limitations of self-assessment and automated consultations features, particularly how they should be used alongside professional care.

### Strengths and limitations

This study has several strengths. It provides a comprehensive view of digital health engagement by examining information-seeking behaviour, multi-platform use, verification practices, and the use of official digital health services, all within a single framework. In addition, the inclusion of both structured questions and open-ended responses allowed for a more nuanced understanding of students’ behaviours and experiences. The questionnaire was pilot tested prior to data collection, which supported clarity and improved the overall quality of the instrument.

However, some limitations should be considered. The cross-sectional design does not allow conclusions about causal relationships. The study did not include longitudinal follow-up; therefore, changes in students’ digital health behaviours over time could not be assessed, and the observed behaviours cannot be attributed to the COVID-19 pandemic. The use of convenience sampling and online recruitment may have introduced self-selection bias, as students with greater interest in or engagement with digital health information may have been more likely to participate. This may have resulted in a sample that was more digitally engaged than the broader student population, limiting the extent to which the findings can be generalized to all university students in Saudi Arabia. Data were collected through a self-administered online questionnaire, which may be subject to recall bias and social desirability bias. Furthermore, some measures were based on self-reported perceptions, which may not fully reflect actual behaviour, and the study did not include an objective assessment of the accuracy of participants’ evaluations or decision-making. The questionnaire assessed behaviours and perceptions relevant to digital health literacy rather than using a validated multidimensional scale to measure the construct comprehensively. More broadly, the six-construct measurement model did not reach conventional model-fit thresholds even after removing three poorly performing items (CFI = 0.893, TLI = 0.875, RMSEA = 0.074), and the verification behaviour and health information-seeking behaviour constructs showed weak convergent validity (AVE = 0.37 and 0.47, respectively), alongside weak or negative factor loadings for some items within the perceived safety and perceived usefulness constructs. Findings involving these constructs should therefore be interpreted cautiously, and future studies should substantively revise or drop the weakest-performing items PU5, PS3, and PS4 in particular, whose wording (in the case of PS4) mixes a safety judgement with a behavioural preference before re-validating the scale. In addition, the structural relationships originally hypothesised between familiarity/awareness and both health information-seeking and self-diagnosis behaviour were not supported by the observed data; the structural model reported in the Results is therefore a post-hoc, data-derived model rather than a confirmatory test of a pre-specified framework, and its path coefficients should be interpreted as exploratory rather than confirmed. Exposure to misinformation was not directly quantified; references to misinformation therefore reflect participants’ reported concerns rather than a measured level of exposure. Given these limitations, the findings should be regarded as exploratory and confirmed in future studies using more representative samples, longitudinal designs, and more robustly validated measures.

## Data Availability

The raw data supporting the conclusions of this article will be made available by the authors, without undue reservation.
