# Supplementary material for: Exploring E-health-literacy-related behaviours and information-seeking practices among university students in Saudi Arabia: a 2024 cross-sectional survey
Source: Front Public Health. 2026 Oct 28;14:1893795. doi: 10.3389/fpubh.2026.1893795 (PMC13752048; doi:10.3389/fpubh.2026.1893795)
Supplement: Supplementary file 1 [file Data_Sheet_1.PDF]

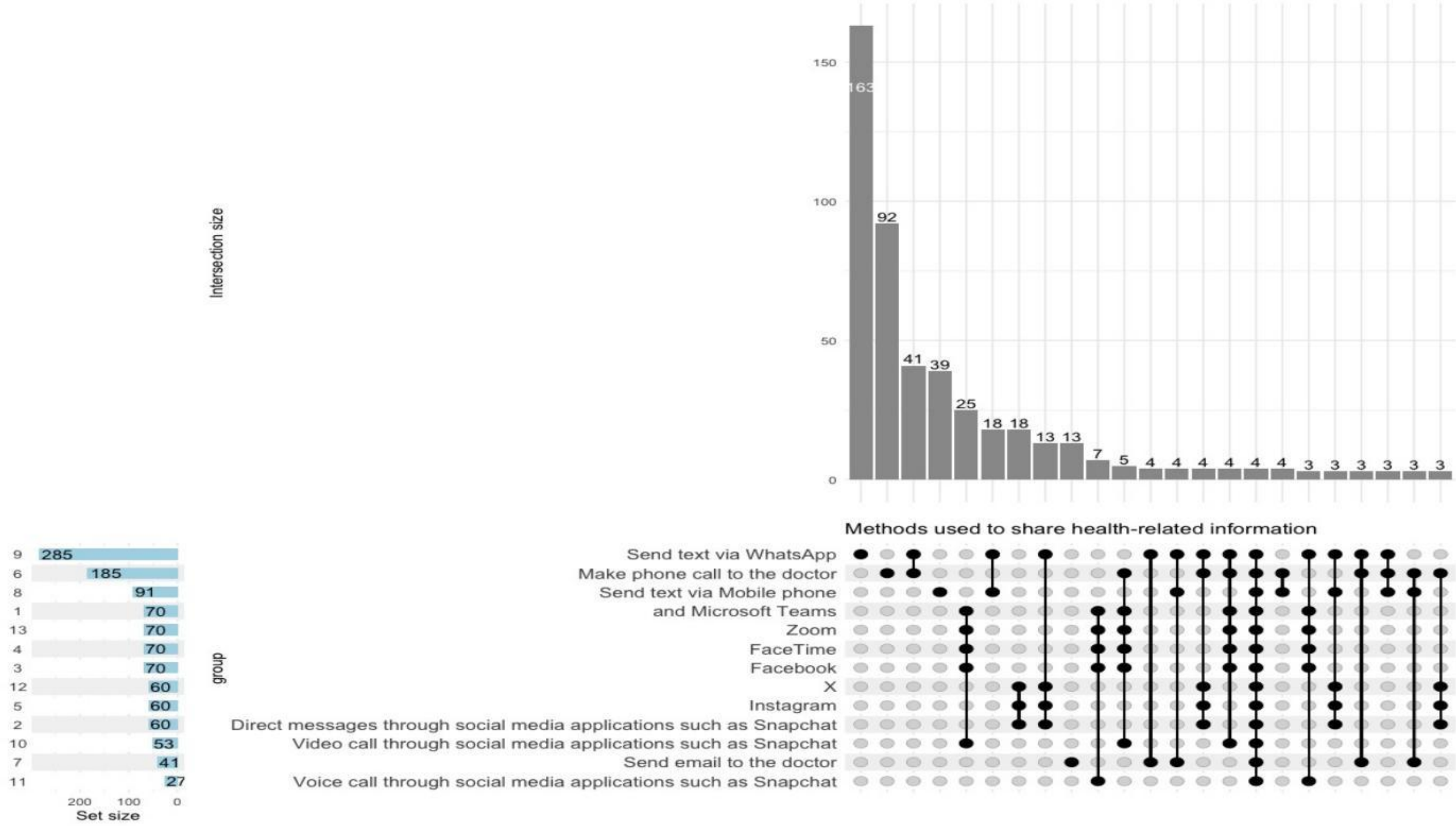

Figure 1: UpSet plot showing combinations of communication methods used by participants to share health-related information through digital and social media channels.

Methods Used to Share Health-Related Information

Figure 1 presents the distribution of health information sharing methods. Text messaging was the most frequently reported sharing channel (n = 285), followed by phone calls to physicians (n = 185). Video-based sharing was the least used method (n < 60). The largest intersection in the UpSet matrix combined text messaging and phone calls to physicians (n = 41), followed by several smaller intersections combining two or more methods (n = 18-25). Multi-method sharing was more common than reliance on a single channel.

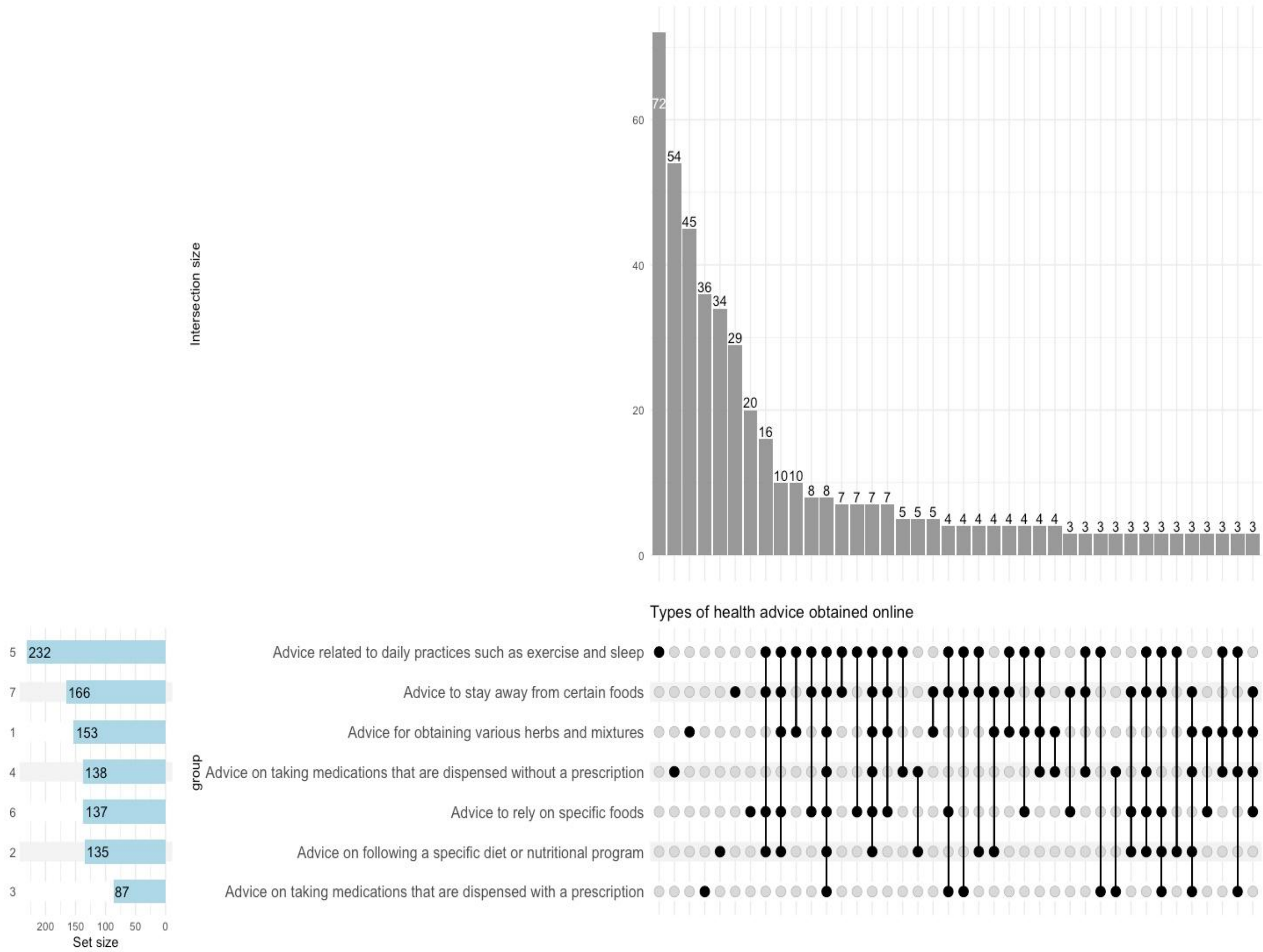

Figure 2: UpSet plot illustrating combinations of health advice and digital health content shared among participants across online and social media environments.

Types of Health Advice Obtained Online

Figure 2 presents the distribution of health advice categories sought online. Lifestyle-related advice covering exercise, sleep, and daily activities was the most frequently obtained category (n=232 respondents), followed by dietary advice (n=166). Non-prescription medication information was reported by approximately 138 respondents, and prescription medication queries by approximately 87 respondents. The intersection analysis identified the combination of lifestyle and dietary advice as the most common co-sought pairing (n=72), followed by various dietary-lifestyle combinations (n = 29-45). Intersections involving medication information alongside lifestyle or dietary advice were smaller (n = 10-20), and complex combinations involving three or more advice types were present at low frequencies (n = 3-8).
